# Mechanisms contributing to persistently activated cell phenotypes in pulmonary hypertension

**DOI:** 10.1113/JP275857

**Published:** 2018-08-07

**Authors:** Cheng‐Jun Hu, Hui Zhang, Aya Laux, Soni S. Pullamsetti, Kurt R. Stenmark

**Affiliations:** ^1^ Department of Craniofacial Biology, School of Dental Medicine University of Colorado Anschutz Medical Campus Aurora CO USA; ^2^ Cardiovascular Pulmonary Research Laboratories, Departments of Pediatrics and Medicine University of Colorado Anschutz Medical Campus Aurora CO USA; ^3^ Department of Lung Development and Remodeling Max Planck Institute for Heart and Lung Research, member of the German Center for Lung Research (DZL) Bad Nauheim Germany; ^4^ Department of Internal Medicine, Universities of Giessen and Marburg Lung Center (UGMLC), member of the DZL Justus‐Liebig University Giessen Germany

**Keywords:** pulmonary hypertension, epigenetic regulators, chromatin structure, Transcription factors

## Abstract

Chronic pulmonary hypertension (PH) is characterized by the accumulation of persistently activated cell types in the pulmonary vessel exhibiting aberrant expression of genes involved in apoptosis resistance, proliferation, inflammation and extracellular matrix (ECM) remodelling. Current therapies for PH, focusing on vasodilatation, do not normalize these activated phenotypes. Furthermore, current approaches to define additional therapeutic targets have focused on determining the initiating signals and their downstream effectors that are important in PH onset and development. Although these approaches have produced a large number of compelling PH treatment targets, many promising human drugs have failed in PH clinical trials. Herein, we propose that one contributing factor to these failures is that processes important in PH development may not be good treatment targets in the established phase of chronic PH. We hypothesize that this is due to alterations of chromatin structure in PH cells, resulting in functional differences between the same factor or pathway in normal or early PH cells *versus* cells in chronic PH. We propose that the high expression of genes involved in the persistently activated phenotype of PH vascular cells is perpetuated by an open chromatin structure and multiple transcription factors (TFs) via the recruitment of high levels of epigenetic regulators including the histone acetylases P300/CBP, histone acetylation readers including BRDs, the Mediator complex and the positive transcription elongation factor (Abstract figure). Thus, determining how gene expression is controlled by examining chromatin structure, TFs and epigenetic regulators associated with aberrantly expressed genes in pulmonary vascular cells in chronic PH, may uncover new PH therapeutic targets.

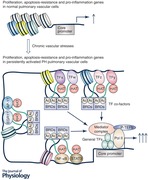

## Introduction

Pulmonary hypertension (PH) exists both as a primary pulmonary vascular disease, as in pulmonary arterial hypertension (PAH) or secondary to an underlying disease, such as chronic exposure to hypoxia as seen in respiratory diseases (COPD, sleep disordered breathing, and others) or chronic exposure to high altitude (Stenmark *et al*. 2009; Stenmark & Rabinovitch, 2010; Simonneau *et al*. 2013). Despite likely differences in the initiating vascular stresses and signalling pathways between primary and secondary forms of PH, there are shared consequences such as vasoconstriction and pathological remodelling of pulmonary vessels that increase pulmonary vascular resistance and stiffness, which stresses the right ventricle (RV), leading to progressive heart failure and death. At the cellular level, pathological remodelling of pulmonary vessels is due to endothelial dysfunction, as well as dysregulated proliferation, apoptosis and inflammatory signalling in all pulmonary vascular wall cells. Importantly, PH vascular cells established from animals with severe hypoxia‐induced PH, as well as humans with end‐stage idiopathic PAH, maintain *in vitro* their dysregulated or persistently activated cell phenotypes such as hyper‐proliferation, apoptosis‐resistance and pro‐inflammation (Li *et al*. 2011; Pullamsetti *et al*. 2016, 2017; Stenmark *et al*. 2018). These activated phenotypes are likely due to a persistently high expression of genes such as *CCND1*, *CCNA2* (hyper‐proliferation), *BCL2*, *BCL2L1* and *Survivin* (apoptosis‐resistance), *CCL2*, *CXCL12*, *GM‐CSF*, *IL6* and *VCAM1* (pro‐inflammation), epidermal growth factor receptor (EGFR) ligands (*AREG*, *EREG*, *TGFA*), fibroblast growth factor receptor (FGFR) ligands (*FGF7*) and MET ligand (*HGF*) (Li *et al*. 2016; Pullamsetti *et al*. 2016, 2017)(authors unpublished observations). These persistently activated phenotypes and aberrant gene expression programmes are maintained *in vitro*, even after multiple passages and in the absence of complex *in vivo* environments, indicating that the gene expression and cell phenotypes of vascular cells of chronic PH are stable and irreversible. Interestingly, all currently approved treatments for PH are based on the “vasoconstrictor hypothesis” of PH and are directed at either inhibiting vasoconstriction (endothelin receptors) or stimulating vasodilatation (prostacyclin and inhibition of phosphodiesterase 5 (PDE5)). Clearly treatments such as prostanoids, endothelin receptor antagonists, phosphodiesterase 5 inhibitors, soluble guanylate cyclase stimulants or, rarely, certain calcium channel blockers can improve patients’ symptoms and extend life (Maron & Galie, 2016; Lau *et al*. 2017). However, though useful, these existing therapies do not halt or reverse PH since these treatments do not directly address the aberrant gene expression programmes responsible for persistently activated cell phenotypes. Thus, it is important to determine the molecular mechanisms that contribute to persistent activation of signalling pathways in cells of the chronically hypertensive vessel wall. Most current studies aimed at identifying therapeutic targets that may reverse the activated cell phenotypes have used an approach aimed largely at determining the initiating signals and their downstream effectors that are important in PH development. These studies have led to the generation of a large number of compelling PH treatment targets, which will be summarized. We will then summarize the success and failure of the clinical trials that target the factors or pathways that are important for PH development. Then, in the following sections, we will describe the data supporting a new hypothesis and its translational implications regarding mechanisms that contribute to the persistently activated cell phenotype that occurs in chronic PH. We specifically propose that alterations in chromatin structure and epigenetic regulators in chronic PH regulate the phenotypes of specific vascular cell types, distinct from the transcriptional mechanisms involved in disease onset.

## Essential role of cell signalling ligands/receptors, signalling transducers, transcription factors, transcription factor co‐factors and epigenetic regulators, in PH onset and development

**Figure 1:**
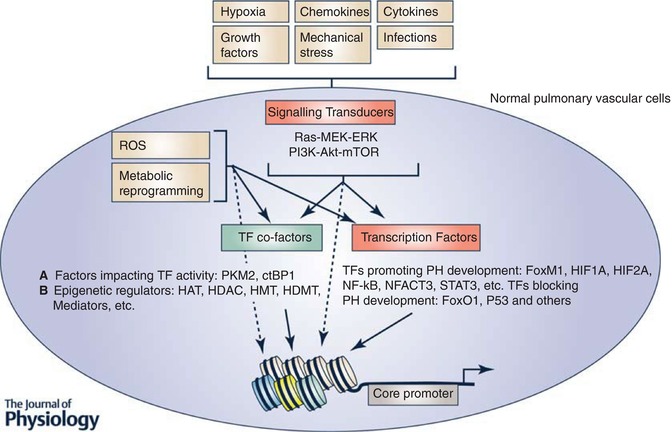
Role of cell signals, signalling receptors, signalling transducers, TFs and TF co‐factors in altering chromatin structure and gene expression in normal pulmonary vascular cells The traditional view is that microenvironmental signals impact gene expression by regulating the activities of TFs that regulate gene expression and disease progression. Studies in the last two decades support roles of extra‐ and intracellular signals in regulating the activities of TF co‐factors (including epigenetic regulators) and nucleosome histone modifications, in addition to regulating TF activity, all of which together control chromatin structure and gene expression.

We have learned a great deal about PH, particularly the pathways or factors that are important in PH onset and development. This knowledge has been derived from studies using epidemiological investigation, animal models and tissues/cells established from human PAH patients. Collectively, these studies support the well‐accepted concept that PH is a multifactorial disease, which can be induced by numerous stimuli and pathological conditions that result in activation of numerous specific signalling pathways and resultant alterations of gene expression (Fig. 1). Below, we will briefly summarize these findings in the order of signalling transduction (from extracellular ligands to signalling transducers), TFs, TF co‐regulators and epigenetic regulators.

### Role of environmental or pathological stimuli in PH development

Environmental or pathological stimuli such hypoxia, mechanical stress, growth factors, chemokines, cytokines, oxidative stress and metabolic reprogramming can all lead to activation of specific signalling pathways resulting in pulmonary vascular cell proliferation, inflammatory response and pulmonary vessel occlusion (Hassoun *et al*. 2009; Stenmark & Rabinovitch, 2010; Schermuly *et al*. 2011; Pullamsetti *et al*. 2016, 2017). Some of these stimuli, such as growth factors, chemokines and cytokines initiate their function by binding to specific receptors located on the cell membrane. That increased expression of growth factors such as platelet‐derived growth factor (PDGF), EGFR ligands, and transforming growth factor β (TGF‐β) are important in initiating development of PH is evidenced by the studies demonstrating that the inhibitor of the EGFR/PDGF receptor downstream effector RAS/RHOB, Tipifarnib (Duluc *et al*. 2017), the TGF‐β ligand trap, a soluble TGF‐β type II receptor extracellular domain expressed as an immunoglobulin‐Fc fusion protein (TGFBRII‐Fc) (Yung *et al*. 2016) and direct PDGF inhibition with the tyrosine kinase inhibitor Imatinib (Pullamsetti *et al*. 2012) can all attenuate PH. In contrast, decreased signalling through the bone morphogenetic protein receptor type II (BMPR2) pathway also leads to PH development (Morrell *et al*. 2006; Guignabert *et al*. 2017; Orriols *et al*. 2017). The role of inflammatory cells, cytokines or chemokines in PH development has also been demonstrated as blocking bone‐marrow‐derived cell recruitment to the lung (Hayashida *et al*. 2005; Frid *et al*. 2006; Gambaryan *et al*. 2010), inhibition of the chemokine SDF‐1 (Young *et al*. 2009), or its receptor CXCR4 (Yu & Hales, 2011), can also block or attenuate PH development (Rabinovitch *et al*. 2014; Pugliese *et al*. 2015). Both intracellular and extracellular redox status has also been shown to contribute to PH development. For instance, the levels of reactive oxygen species (ROS) are increased under hypoxia, due to increased production of ROS from mitochondria complex II and/or complex III (Paddenberg *et al*. 2003; Guzy *et al*. 2007). The role of ROS in PH is supported by the spontaneous development of PH in mice in which superoxide dismutase (*Sod1*) is deleted in smooth muscle cells (SMCs) (Nozik‐Grayck *et al*. 2014) while hypoxia‐induced PH is significantly attenuated in mice with overexpression of extracellular SOD (EC‐SOD) in the lung (Nozik‐Grayck *et al*. 2008).

### The role of signalling transducers in PH development

Association of ligands such as growth factors, cytokines, chemokines, or extracellular matrix to their receptors often leads to activation of intracellular and/or membrane‐associated protein kinases, which results in signal transduction and signal amplification. Here, we primarily focus on the role of Ras–MEK–ERK and PI3K–Akt–mTOR signalling transducers in PH as these factors play critical roles in cell proliferation, survival and motility. Activation of Ras proteins, which, in turn, transduces signals through Raf, MEK and ERK, can be mediated by growth factors and extracellular matrix‐mediated signals. Specific to PH endothelial cells, Ras can also be activated by BMPR2 silencing (Awad *et al*. 2016). The activation of Ras–MEK–ERK is well demonstrated in PH (Lane *et al*. 2005). The function of Ras–MEK–ERK in PH development is supported by the fact that *Raf‐1* kinase inhibitor protein knockout mice exhibit more severe hypoxia‐induced PH (Morecroft *et al*. 2011). The PI3K–Akt–mTOR pathway is often activated by receptor tyrosine kinases, G protein‐coupled receptors and integrins. Multiple studies have documented the role of PI3K–Akt–mTOR in PH initiation including attenuated development of hypoxia‐induced PH in rats when treated with PI3K or Akt inhibitors (Garat *et al*. 2013), or in mice with SMC‐specific deletion of *Akt* (Tang *et al*. 2015) while knockdown of *PTen*, a negative regulator of Akt activation, leads to spontaneous PH (Nemenoff *et al*. 2008).

### The roles of TFs in PH development

The activities of transcription factors (TFs) are often regulated by signal transducers initiated from outside of the cell but they can also be modulated by intracellular signals. Regulation of TF activity by signalling molecules is typically mediated through post‐translational modifications such as phosphorylation, acetylation and methylation, resulting in alterations of TF protein stabilization, translocation between cytoplasm and nucleus, alteration of TF binding affinity to its co‐activators, and alteration of TF binding to DNA (Spitz & Furlong, 2012; Bhagwat & Vakoc, 2015). Multiple TFs have been implicated in PH development (Pullamsetti *et al*. 2016). For example, hypoxia‐inducible factors (HIFs) are key regulators of the molecular response to hypoxia. The target genes of HIFs include genes controlling neovascularization, cell proliferation, migration, metabolism and others (Pawlus & Hu, 2013). Studies from multiple laboratories using mouse models have established a critical role of HIF2 in hypoxia‐mediated PH in which global reduction (Brusselmans *et al*. 2003) or knockout of *Hif2* in endothelial cells (ECs) (Bryant *et al*. 2016; Tang *et al*. 2018) or in pulmonary ECs (Cowburn *et al*. 2016) reduces or completely blocks the development of PH. Conversely, activation of HIF2 via inactivating mutation of Von Hippel‐Lindau (*Vhl*) (Hickey *et al*. 2010) or deletion of *Phd2* (Dai *et al*. 2016; Kapitsinou *et al*. 2016; Wang *et al*. 2016; Tang *et al*. 2018), or activating mutation of *Hif2a* (Tan *et al*. 2013) leads to PH development under normoxic conditions. Increased expression of *FOXM1*, a transcription factor crucial for G1–S and G2–M cell cycle progression and ROS‐induced DNA damage repair has been found upregulated in PH and blocking its expression prevents and reverses hypoxia‐induced PH in rodents (Bourgeois *et al*. 2018; Dai *et al*. 2018). PH development is often associated with early and persistent perivascular inflammation in animal models of PH (Li *et al*. 2011; Stenmark *et al*. 2012) and persistent inflammation is also observed in most chronic forms of human PH (Tuder *et al*. 2013; Rabinovitch *et al*. 2014; Ghataorhe *et al*. 2017). Increased activation of inflammatory TFs such as STAT3 (Paulin *et al*. 2011a,b, 2012) and nuclear factor κB (NF‐κB) (Sawada *et al*. 2007; Huang *et al*. 2008; Kimura *et al*. 2009; Hosokawa *et al*. 2013; Price *et al*. 2013; Farkas *et al*. 2014; Li *et al*. 2014) have been consistently observed in animal models and human PH. Further, STAT3 and NF‐κB inhibition either block or attenuate PH development since these TFs not only sustain inflammatory responses but also promote cell proliferation, survival and metabolic reprogramming (Grivennikov & Karin, 2010). Further, inhibition of a TF called NFATc3, that is activated by increased levels of ROS, prevents hypoxia‐induced PH in mice (Ramiro‐Diaz *et al*. 2013).

Reduced activities of TFs such as p53 and FoxOs also promote PH development. p53 is necessary for responding to DNA damage and other stresses, and p53 activation often leads to inhibition of cell proliferation. Thus, it is not surprising that reduced p53 expression/activity contributes to PH development in which more severe PH is observed in *Tp53* knockout mice under chronic hypoxia (Mizuno *et al*. 2011) or in rats treated with a p53 inhibitor (Jacquin *et al*. 2015). The activities of FoxO TFs are often reduced by growth factors and inflammatory cytokine‐mediated signalling pathways, leading to increased cell proliferation, survival and metabolic reprogramming. Indeed, both *in vitro* and *in vivo*, reduction of FoxO activity increases the severity of PH while restoration of FoxO activity can block or reverse PH (Savai *et al*. 2014).

### The role of TF co‐factors in PH development

A TF co‐factor (co‐activator or co‐repressor) is a type of protein that itself has no DNA binding activity, but can interact with other general or sequence‐specific TFs to modify the ability of TFs to regulate gene expression. Broadly speaking, TF co‐factors can be divided into two types, one with activity on chromatin structure, called epigenetic regulators, and another type functioning on TF activity only (Kornberg, 2001; Cosma, 2002; Ries & Meisterernst, 2011). We first focus on two non‐epigenetic TF co‐factors: PKM2 and C‐terminal binding protein‐1 (CtBP1). Both of these factors are controlled by the metabolic state of the cell, which in all pulmonary vascular wall cells is known to change in both acute and chronic forms of PH (Sutendra & Michelakis, 2014; Stenmark *et al*. 2015; Plecita‐Hlavata *et al*. 2016, 2017; D'Alessandro *et al*. 2018). The metabolic adaptation, often referred to as ‘Warbug‐like” leads to increased glycolysis and increased fatty acid oxidation, but reduced oxidative phosphorylation in mitochondria. The reduced oxidative phosphorylation in mitochondria is the result of reduced input of acetyl‐CoA to TCA, and/or increased mitochondria fission (D'Alessandro *et al*. 2018). *PKM2* is one of the splicing isoforms of a gene called pyruvate kinase muscle type, a gene that plays an important role in glycolysis (Wong *et al*. 2015; Dayton *et al*. 2016; Dong *et al*. 2016). However, in addition to its role in glycolysis, PKM2 can serve as a HIF1 co‐activator by promoting HIF1's role in activating HIF target genes through its binding to and phosphorylation of HIF1α protein (Luo & Semenza, 2011; Luo *et al*. 2011). PKM2 also has other functions including activating cell proliferation via phosphorylation of the cell cycle regulator BUB3 and regulating chromatin structure by phosphorylating histones (Dong *et al*. 2016). Indeed, inhibition of PKM2 activity directly, or of its upstream activator or downstream effectors reduce the “activated” phenotypes of PH vascular cells (Caruso *et al*. 2017; Zhang *et al*. 2017). Different from PKM2, CtBPs function as transcriptional corepressors (Kuppuswamy *et al*. 2008; Wang *et al*. 2012; Blevins *et al*. 2017). CtBPs repress gene expression by binding to an inhibitory TF and recruiting histone‐modifying enzymes that add repressive histone marks and remove activating histone marks (Byun & Gardner, 2013). In PH fibroblasts, CtBP activity is increased, due to increased free NADH (increased NADH/NAD^+^ ratio). Increased CtBP activity enhances cell proliferation and apoptosis resistance by decreasing expression of cell cycle inhibitors such as p15 and p21 and pro‐apoptosis genes such as *NOXA* and *PERP* (Li *et al*. 2016). Importantly, normalizing metabolic activity via metabolic inhibitors such as 2‐deoxyglucose (2DG) or directly reducing CtBP1 expression reduces PH fibroblast proliferation and apoptosis resistance (Li *et al*. 2016).

### The role of epigenetic regulators in PH development

Another type of TF co‐factors in eukaryotic cells are chromatin or epigenetic regulators that function in gene expression by controlling chromatin structure (Shlyueva *et al*. 2014; Voss & Hager, 2014). These factors include histone post‐translational modifying enzymes such as histone acetylases (CREB‐binding protein (CBP) and p300), readers of histone modifications such as bromodomain (BRD) proteins, Brahma‐associated factor (BAF) complex, Mediator complexes and others (Fig. 1). All of these epigenetic regulators can be recruited by TFs, but can also be additionally recruited by other chromatin‐associated proteins including histones (see below). CBP and its paralogue p300 are histone acetyl‐transferases (HATs) that acetylate histones at both promoters and enhancers as well as numerous non‐histone proteins including TFs (Spange *et al*. 2009; Slingerland *et al*. 2014). HATs are often recruited to chromatin by TFs. BRD4 is a member of the BET (bromodomain and extra‐terminal domain) family proteins that are characteristic of two tandem bromodomains (BDs) located in the N‐terminus. The BDs of BET proteins recognize acetylated‐lysine residues in nucleosomal histones and other proteins such as TFs (Filippakopoulos *et al*. 2012). BRD proteins can activate gene transcription by recruiting positive transcription elongation factor (P‐TEFb), Mediator, and other chromatin remodelling complexes including BAF complex (Jang *et al*. 2005). Mediator is a large multiprotein complex >1 MDa in size and >30 nm in length. Besides interacting with BRD proteins, different TFs bind different Mediator subunits. Thus, Mediator complex can act as a bridge mediating interaction between TFs and components of the general TFs (GTFs)/RNA polymerase II (RNA Pol II). Additionally, Mediator also activates gene transcription by recruiting the P‐TEFb to activate elongation activity of RNA Pol II (Allen & Taatjes, 2015). BAF complexes (not shown in Abstract figure), which belong to the SWI/SNF family of ATPase‐dependent chromatin remodelling complexes, are also involved in chromatin structure changes through their effects on movement of nucleosome position relative to specific DNA sequence, ejection of nucleosome, and exchange of classic core histones with variant histones (Halliday *et al*. 2009; Reisman *et al*. 2009). BAF complexes can be recruited by TFs, BRDs and acetylated histones since BAF complex contains multiple bromodomain‐containing proteins (Halliday *et al*. 2009; Reisman *et al*. 2009).

Recently, new work has led to an appreciation of the important role epigenetic regulators play in PH initiation as several epigenetic regulators such as histone deacetylases (HDACs), and double bromodomain proteins (BRDs), have been shown to exhibit increased expression in PH vascular cells (Zhao *et al*. 2012; Meloche *et al*. 2015, 2017). Furthermore, HDAC and BRD inhibitors have been shown to prevent or reverse PH (Zhao *et al*. 2012; Meloche *et al*. 2015, 2017).

## Can we reverse the persistently “activated” phenotypes of PH vascular cells, based on potential targets uncovered in PH initiation studies?

There is a large body of compelling data, including some that was described above, supporting critical roles for factors ranging from membrane receptors, signalling transducers, TFs, non‐epigenetic TF co‐factors, and epigenetic regulators in PH development (Fig. 1). Clearly, the alterations of these factors or pathways are required in PH development, and are at least in part, responsible for changing the gene expression programme that gradually transforms the normal pulmonary vascular cells to the activated PH vascular cells (Abstract figure). Importantly, the role of many of these factors has been evaluated with regard to both PH prevention and PH reversal in animal models. Further, the function of these factors, in some cases, has also been demonstrated in one or multiple human PH vascular cells *in vitro*. Thus, these studies provided a large list of potential new PH treatment targets (Lythgoe *et al*. 2016; Wilkins, 2018). However, despite all these efforts, there has been little success in new therapies targeting structural remodelling or the activated phenotypes of the PH vascular cells (Lythgoe *et al*. 2016; Wilkins, 2018). For example, drugs such as Terquride (a serotonin antagonist), statins and vasoactive intestinal peptide (VIP), all with very promising effects in pre‐clinical animal models (Said *et al*. 2007; Morecroft *et al*. 2010; Wright *et al*. 2011), all failed to meet their primary endpoint in clinical trials. Further, the tyrosine kinase inhibitor Imatinib, though shown to improve haemodynamics in many patients, has not been licensed because of unacceptable side effects (Lythgoe *et al*. 2016). More recent reports indicate that an inhibitor for ASK1 (apoptosis signal‐regulating kinase 1) also failed to meet the primary endpoint in a clinical trial (Wilkins, 2018), again despite the demonstrated critical role of ASK1 in PH animal models and in human PH vascular cells (Welsh *et al*. 2001; Mortimer *et al*. 2007; Church *et al*. 2015; Budas *et al*. 2018). Additionally, Eiger BioPharmaceuticals announced that it has halted clinical development of Ubenimex for PAH due to lack of efficacy to treat PH in the Phase 2 LIBERTY study although leukotriene B4, the target of Ubenimex, plays an important role in PH in animals (Tian *et al*. 2013). Collectively, failure of these clinical trials indicates a significant challenge in developing new PH treatments. The reasons for these disappointing findings could be multiple, including a need to improve the selection of patients in clinical trials and the poor fidelity of animal models of PH for the human PH disease, all of which have been reviewed by Lythgoe *et al*. (2016). We believe that another factor usually not considered, but that could also contribute to the failure of translation from animal studies to humans, is that most PH targets tested have been uncovered from studies in the early stages of PH development. We think that factors or pathways that are essential in PH development may or may not be critical in established disease due to extensive changes in chromatin structure, between normal pulmonary vascular cells and vascular cells in chronic PH patients (Stenmark *et al*. 2018). There are multiple examples in a variety of cancers that support the hypothesis that pathways/factors that play major roles in cancer development play no or only minor roles in maintaining the transformed phenotypes of established cancers. The best studied example is *KRAS* whose mutation drives multiple early onset lung tumours. Interestingly, these lung cancers often become KRAS independent, if the phenotype of the cancer switches from epithelial to mesenchymal (Singh *et al*. 2009). Further, although mutations of *EGFR* predispose to the development of lung cancer, most of the *EGFR* mutated lung cancers are resistant to EGFR inhibition (Ware *et al*. 2013). Also, both HIF1 and HIF2 are required for initiation of clear cell renal cell carcinoma (ccRCC) (Schonenberger *et al*. 2016). However, in the later stage of human ccRCC tumours, both HIF1 and HIF2 are dispensable (Shen *et al*. 2011; Murakami *et al*. 2017).

Below, we will examine the importance of chromatin structure and epigenetic regulators in maintaining the activated phenotypes of PH vascular cells. Because the cancer paradigm is often invoked in explaining the cellular changes observed in severe chronic PH, we will start this section by summarizing the extensive epigenetic changes observed in cancer cells and reported success of developing epigenetic regulators as promising cancer treatment targets in human cancers.

## Future research in “transcriptional addiction” of PH vascular cells: roles of chromatin structure, multiple transcription factors and epigenetic regulators in persistent activation of PH vascular cells

### Transcriptional addiction as a promising cancer therapeutic strategy

**Figure 2:**
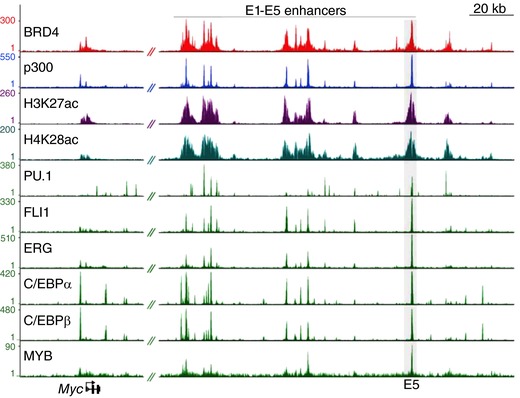
ChIP‐Seq occupancy profiles of epigenetic regulators BRD4 and p300, active histone modification marks H3K27Ac and H4KAc, and TFs PU.1, FL1, ERG, C/EBPα, C/EBPβ and MYB at the *MYC* locus in myeloid leukaemia cells Note there are at least 5 distinct enhancers for *MYC* gene expression in a more than 100 kb regulatory region. Each enhancer exhibits co‐existence of high histone acetylation and high binding densities of several TFs and epigenetic regulators (Roe *et al*. 2015). Importantly, although TFs exhibit unique binding densities to each enhancer, epigenetic regulators exhibit a similar binding pattern to each active enhancer, suggesting that epigenetic regulators are commonly required for different TFs to regulate gene expression.

Research performed in the last 20 years makes it clear that mutated signalling and TFs that initiate cancer development often end in changes of chromatin structure and gene expression in cancer cells (Lee & Young, 2013; Sur & Taipale, 2016; Bradner *et al*. 2017) since kinases can alter chromatin structure by: (1) controlling the activities of epigenetic regulators; (2) controlling chromosomal histone phosphorylation; and (3) controlling the levels of epigenetic regulators on chromatin by regulating the activities of TFs (Badeaux & Shi, 2013; Morgan & Shilatifard, 2015; Sur & Taipale, 2016) (Fig. 1). For example, expression of oncogenes such as *MYC* are much higher in pancreatic and colorectal cancers, as well as in T cell leukaemia, *versus* their normal control cells (see Fig. 6*B* of Hnisz *et al*. 2013). Interestingly, detailed analysis of *MYC* enhancers in myeloid leukaemia cells indicates that the *MYC* gene is regulated by at least five active enhancers (E1–E5) that cover a large region (more than 100 kB) of DNA and each enhancer exhibits high levels of active histone modification marks (H3K27Ac and H4K8Ac), high binding densities of epigenetic regulators (BRD4 and p300) and multiple TFs (PU.1, FLI, ERG, CEBPα, CEBPβ and MYB) (Roe *et al*. 2015) (Fig. 2), all of which are marks of open chromatin structure. Further, all the BRD4‐occupied sites overlap with binding sites of one or more TFs and most BRD4‐enriched regions exhibit binding of several TFs (Fig. 2), indicating BRD4 protein is commonly required for different TFs to regulate gene expression. The role of epigenetic deregulation and chromatin structure changes in cancer gene expression is also supported by the fact that almost every cancer cell type contains mutation of genes involved in epigenetic regulation (Koschmann *et al*. 2017). All these studies support a critical role of chromatin structure and epigenetic regulators in cancer development and maintenance (Badeaux & Shi, 2013). Thus, epigenetic regulators such as histone methyltransferases and de‐methylases, histone acetylases and deacetylases, and the BET proteins (BRD2, BRD3 and BRD4) are attractive targets for therapeutic intervention in cancers (Barbieri *et al*. 2013; Heerboth *et al*. 2014; Slingerland *et al*. 2014; Wee *et al*. 2014; Cai *et al*. 2015; McGrath & Trojer, 2015; Jones *et al*. 2016). Clearly, like cancer, there is a “transcriptional addiction” in PH vascular cells, reflected in persistently high expression of genes involved in hyper‐proliferation, apoptosis‐resistance and pro‐inflammation (Li *et al*. 2016; Pullamsetti *et al*. 2016, 2017). We have preliminary data to support a hypothesis that high expression of genes involved in the persistently activated pro‐inflammatory phenotype of PH vascular cells, at the chromatin level, are maintained by an open chromatin structure and multiple TFs, via the recruitment and maintenance of high levels of epigenetic regulators such as histone acetylate P300/CBP, histone acetylation readers including BRDs, Mediator complex and positive transcription elongation factor (Abstract figure). Thus, it will be important to perform chromatin immunoprecipitation‐sequencing (ChIP‐Seq) occupancy profiles of histone modifications, TFs and epigenetic regulators in PH vascular cells, as has been done for oncogenes in cancers (Fig. 2).

### Mechanisms that can drive “transcription addiction” of PH vascular cells

We believe that various components of transcriptional and epigenetic control play a crucial role in controlling gene expression in chronic disease such as PH, in which mutations of epigenetic regulators are rarely reported. In addition, we also posit that the components that control gene expression in normal and persistently activated PH vascular cells are different. Thus, determining the mechanisms controlling gene expression in persistently activated PH vascular cells may uncover new molecular mechanisms and may form the basis on which novel PH therapeutic targets will be developed.

The genomic DNA in eukaryotic cells is packaged in the nucleosome, which consists of two copies of each histone protein (H2A, H2B, H3 and H4) and 146 base pairs of superhelical DNA wrapped around this histone octamer. The nucleosome structure creates a problem for TFs and RNA polymerase to access the DNA, but also provides an opportunity for regulated gene expression. It is now accepted that the level or rate of gene transcription in eukaryotic cells is determined by interplay among *cis*‐acting regulatory DNA elements, which includes the core promoter, proximal promoter regions as well as those that act over large genomic distances, such as enhancers (Spitz & Furlong, 2012), and *trans*‐acting factors including gene‐specific TFs, epigenetic regulators, general TFs and RNA polymerase II. Thus, it is important to address all three components (chromatin structure, TFs and epigenetic regulators) that control gene expression in PH vascular cells.

### Determine the chromatin structure of genes involved in the persistently activated cell phenotypes of PH vascular cells

**Figure 3:**
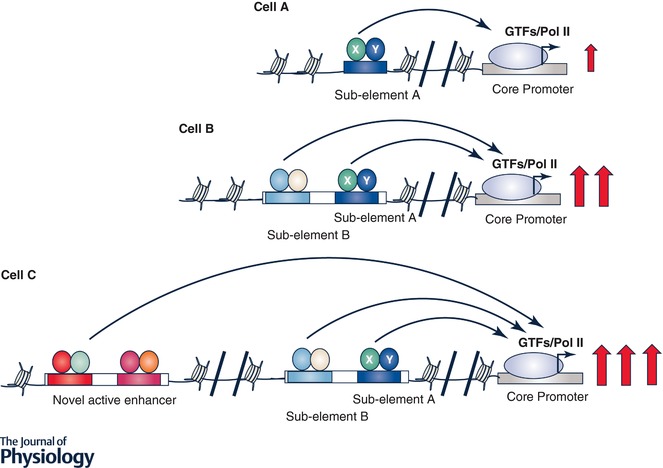
Hypothetical representation of chromatin structure determines the functional importance of TFs TFs X and Y play a critical role in expression of this gene in cell A by binding to sub‐element A. But a more “open” chromatin structure, allowing other TFs to bind to the regulatory elements of this gene, diminishes the contribution of TFs X and Y in expression of this gene in cells B and C.

Enhancers, composed of dense clusters of TF binding motifs, are cell type‐specific and highly regulated. Thus, enhancers are critically important in controlling a subset of eukaryotic genes, called regulated genes, that are often involved in development, cell identify and functional phenotypes (Shlyueva *et al*. 2014; Smith & Shilatifard, 2014; Heinz *et al*. 2015). Enhancer DNA can exist in an active (accessible to TF binding) or inactive (inaccessible) status. Tools now exist for annotating the status of the enhancers on a genome‐wide scale by measuring levels of histone modifications, TF and epigenetic regulator binding and chromatin accessibility. These approaches have shown that the functional enhancer landscape is largely unique to each cell type and maintained by lineage‐specific TFs and epigenetic regulators. However, new evidence reveals how acute or chronic signalling events can lead to reprogramming of enhancer configurations (Brown *et al*. 2014; Lavin *et al*. 2014). Studies have uncovered multiple mechanisms involved in reprogramming enhancers during development and disease progression. Regulation of gene expression is inherently associated with alterations in chromatin architecture because TFs often recruit co‐activators such as p300 to acetylate histones at the enhancer at which the TF binds, but such histone acetylation often extends to neighbouring nucleosomes, leading to larger active DNA regions and more active enhancers. These transient histone modifications to larger DNA regions are heritable if cells are proliferating (Probst *et al*. 2009). It is well accepted that PH development involves extensive vascular cell proliferation at least at the peak stage(s) of PH development, thus transient increased expression of genes involved in hypoxia response, inflammation, growth factor signalling and others, in a combination of cell proliferation, may lead to a more “open” chromatin structure of these genes. The second way of TF‐mediated enhancer reprogramming is mediated by a subset TFs called ‘pioneer factors’. These pioneer TFs are particularly important in creating brand new active enhancers due to their ability to engage silent, closed enhancers (Zaret & Carroll, 2011; Adam *et al*. 2015). Studies have identified about 100 pioneer TFs in development and cancer research. While clearly functionally important, so far the pioneer TF concept has not been introduced into the PH research. While TF‐mediated enhancer reprogramming is well accepted at least in development and cancer research, new evidence supports a direct role of cell signalling in reprogramming enhancers by kinase‐mediated phosphorylation of histones and/or epigenetic regulators (Badeaux & Shi, 2013). Also, the activity of chromatin regulators can be altered by other mechanisms such as metabolic intermediates and redox stress (Berger & Sassone‐Corsi, 2016; Kreuz & Fischle, 2016; Reid *et al*. 2017). There is extensive literature demonstrating alterations in growth factor‐mediated signalling, reprogramming of cellular metabolic and redox state during PH progression (Merklinger *et al*. 2005; Schermuly *et al*. 2005; Plecita‐Hlavata *et al*. 2016; Zhang *et al*. 2017), which could directly impact chromatin structure in PH vascular cells. Further, demethylation of H3K4me3 and H3K27me3, two critical histone modification events, is mediated by oxygen and 2‐oxoglutarate dependent dioxygenase enzymes such as demethylase KDM6B/JMJD3, whose function can be inhibited by oxygen deprivation (hypoxia) (Hancock *et al*. 2015; Prickaerts *et al*. 2016). All these studies provide ample support for the hypothesis that there are significant differences in chromatin structure between normal and PH vascular cells. Thus, it is essential to determine the chromatin structure in control and PH vascular cells. Such studies may also provide a molecular explanation of why targeting TFs that are important in PH initiation may or may not be sufficient in PH treatment. For example, in a normal vascular cell (Fig. 3, cell A), TFs X and Y are critical in expression of this gene, but TFs X and Y become less and less important in activation of this gene in both cells B and C (Fig. 3) in which this gene's expression is regulated by additional TFs, including ubiquitous TFs that have no role in regulating this gene in cell A (Fig. 3), due to more open chromatin structure in cells B and C. Chromatin structure analysis will also allow us to identify the set of TFs that are potentially associated with these newly activated enhancers, using motif analysis.

### Determine the identities and function of the TFs that are associated with the genes involved in persistently activated cell phenotypes of PH vascular cells

**Figure 4:**
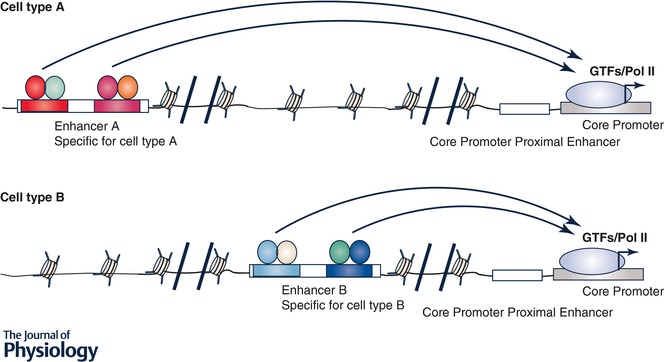
Enhancers are often cell type specific Enhancer A is important in controlling this gene in cell type A while enhancer B is critical in regulating the same gene in cell type B. Thus, the TFs that regulate the same gene could be totally different between cell type A and cell type B. This hypothetical model suggests that targeting a specific TF that is effective in reducing a specific gene in one cell type may have no role in reducing the same gene in another cell type.

TFs play an indispensible role in the control of chromatin structure, gene expression, response to specific signals and thus disease initiation. Due to alterations of the endogenous as well as extracellular microenvironment between normal and diseased tissues/organs, the set of TFs active in normal cells and diseased cells often only partially overlap. Even though the expression and activity of a specific TF may be maintained in established disease, global changes in chromatin accessibility may reprogramme the TF binding profile, thus its function. Further, TF binding to DNA often depends on its partner(s), thus changes in expression/activity of TF binding partners may also alter the function of a specific TF in established disease. TFs can be broadly divided into two types. One is pioneer TFs or lineage‐specific TFs that are important in creating and maintaining cell identity as well as cellular functional phenotypes (Adam *et al*. 2015). Another type of TF are stress TFs such as HIF, STAT3, NF‐κB and activator protein 1 (Ap‐1) that are activated in response to specific signals. Pioneer and stress TFs often work together to regulate gene expression in which pioneer TFs establish the competency for stress TFs to further activate gene expression. TF expression and their activities in diseased cells are rarely studied, due to the misconception that epigenetic changes that are introduced in disease progression are sufficient to maintain gene expression. In fact, studies have shown that the maintenance of chromatin structure and gene expression patterns requires participation of both TF activity and epigenetic regulators (Spitz & Furlong, 2012). Thus, it is critical to determine the set of TFs that function in PH vascular cells to increase our understanding of PH‐activated phenotypes and to provide potential therapeutic targets for PH disease (Bhagwat & Vakoc, 2015). TFs that are critically important for control and PH vascular cells can be profiled by checking their gene expression (using RNA‐Seq) and their binding profile in genomic DNA (using ChIP‐Seq of TF). The functions of TF can be determined using TF specific inhibitors and/or siRNA‐mediated knockdown or CRISPR/cas9‐mediated gene knockout in PH vascular cells. We must emphasize the importance of studying the function of TF in all of the PH vascular cells involved in the disease process. It is well accepted that enhancer landscape is often unique to each cell type, suggesting that different enhancers (Fig. 4), and thus different TFs, could be utilized in different cell types for the same gene.

### Determine the identities and the function of the epigenetic regulators that are associated with the genes involved in persistently activated cell phenotypes of PH vascular cells

Besides chromatin structure and TFs, a third component that is critically important in gene regulation is epigenetic regulators. Several groups including ours have reported the potential for HDAC inhibitors (HDACi) to reverse hypoxic PH and to have beneficial effects on cardiac fibrosis (Cavasin *et al*. 2012; Zhao *et al*. 2012; De Raaf *et al*. 2014; Williams *et al*. 2014). Recently, the Bonnet group has presented evidence for increased BRD4 levels in the SMCs not only in remodelled pulmonary arteries but also in the coronary artery vasculature (Meloche *et al*. 2015, 2017). Importantly, these investigators demonstrated that the BRD inhibitor (BRDi) JQ1 could not only mitigate the hyper‐proliferative pulmonary hypertensive SMC phenotype *in vitro* but could reverse the vascular remodelling observed in the Sugen/hypoxia model (Meloche *et al*. 2015). However, most HDACi and BRDi studies have not addressed the downstream target(s) of the inhibitors. Progressing to clinical trials without understanding the downstream effectors of these inhibitors is premature as we need more information on their prospective gene targets in different cells and at different disease stages (Andrieu *et al*. 2016). Thus, determining the binding profile and function of epigenetic regulators such as HATs (p300), BRDs (BRD4) and HDACs in controlling the persistently activated phenotypes of PH cells such as SMCs, fibroblasts (Fibs), ECs and macrophages is essential. Our preliminary data indicate that inhibitors for epigenetic regulators produce more potent effects on all phenotypes in all PH vascular cells than does inhibition of a single transcription factor, due to their common requirement for gene transcription, independent of the signal types, TFs and cell types. Proteins such as BRDs are particularly interesting, because different from histone modifying enzymes such as HDAC, HAT, methyl‐transferase and demethylase, BRD proteins are only involved in gene transcription while the histone‐modifying enzymes also regulate the activities of non‐histone proteins (Choudhary *et al*. 2009; Spange *et al*. 2009), which have functions beyond gene transcription and make it challenging to understand the downstream targets of these enzymes.

## Conclusion

A root problem in the vascular remodelling observed in chronic PH is the presence of “persistently activated” cell phenotypes with aberrant gene expression. It is likely that currently approved treatments do not directly target this problem. In this review we raise the possibility that one factor that contributes to these failures is that factors/pathways important in PH initiation and development may or may not be good treatment targets later in the disease, due to alterations of chromatin structure in “persistently activated” PH cells. These changes in chromatin can result in distinctly different functional responses to a signalling pathway or TF in normal or early PH cells *versus* cells in chronic PH. We provide evidence that another approach to uncover novel therapeutic targets for established PH is to determine the molecular mechanism(s) controlling gene expression in chronic established PH. Our central hypothesis is that high expression of genes involved in the persistently activated phenotype of PH vascular cells are maintained by an open chromatin structure and multiple TFs via the recruitment and maintenance of high levels of epigenetic regulators such as histone acetylases P300/CBP, histone acetylation readers including BRDs, Mediator complex and positive transcription elongation factor (Abstract figure). The evidence provided for this hypothesis comes at present largely from studies in the cancer field. The data shown regarding how aberrant gene expression is controlled by chromatin structure, TFs and epigenetic regulators may provide potential therapeutic targets for PH treatment. We emphasize that in PH it is not easy to extrapolate the findings from one cell type to other disease‐involved cell types or findings from one pathway to other pathways. Thus, we believe effective treatments for PH must target the phenotypes of excessive proliferation, apoptosis‐resistance, pro‐inflammation, in all or at least most cell types of PH vascular cells such as SMCs, Fibs, ECs and inflammatory cells. Thus, studies of “transcriptional addiction” in PH vascular cells should be performed in all the aforementioned cells and in their most “activated” phenotypic state. Due to cross‐talk among PH vascular cell types, at least some of the studies will need to be performed in co‐culture systems to integrate protein and metabolic cross‐talk. There is reason for excitement regarding potential new treatment options but more knowledge is needed before we proceed.

## Additional information

### Competing interests

None declared.

### Author contributions

All authors have approved the final version of the manuscript and agree to be accountable for all aspects of the work. All persons designated as authors qualify for authorship, and all those who qualify for authorship are listed.

### Funding

K.R.S. is supported by US National Heart, Lung, and Blood Institute grants P01HL014985, T32HL007171 and R01HL114887, and the US Department of Defense grant no.W81XWH‐15‐1‐0280.

## References

[tjp13073-bib-0001] Adam RC , Yang H , Rockowitz S , Larsen SB , Nikolova M , Oristian DS , Polak L , Kadaja M , Asare A , Zheng D & Fuchs E (2015). Pioneer factors govern super‐enhancer dynamics in stem cell plasticity and lineage choice. Nature 521, 366–370. 25799994 10.1038/nature14289 PMC4482136

[tjp13073-bib-0002] Allen BL & Taatjes DJ (2015). The Mediator complex: a central integrator of transcription. Nat Rev Mol Cell Biol 16, 155–166. 25693131 10.1038/nrm3951 PMC4963239

[tjp13073-bib-0003] Andrieu G , Belkina AC & Denis GV (2016). Clinical trials for BET inhibitors run ahead of the science. Drug Discov Today Technol 19, 45–50. 27769357 10.1016/j.ddtec.2016.06.004 PMC5116321

[tjp13073-bib-0004] Awad KS , Elinoff JM , Wang S , Gairhe S , Ferreyra GA , Cai R , Sun J , Solomon MA & Danner RL (2016). Raf/ERK drives the proliferative and invasive phenotype of BMPR2‐silenced pulmonary artery endothelial cells. Am J Physiol Lung Cell Mol Physiol 310, L187–L201. 26589479 10.1152/ajplung.00303.2015 PMC4719048

[tjp13073-bib-0005] Badeaux AI & Shi Y (2013). Emerging roles for chromatin as a signal integration and storage platform. Nat Rev Mol Cell Biol 14, 211–224. 10.1038/nrm3545 PMC4082330 23524488

[tjp13073-bib-0006] Barbieri I , Cannizzaro E & Dawson MA (2013). Bromodomains as therapeutic targets in cancer. Brief Funct Genomics 12, 219–230. 23543289 10.1093/bfgp/elt007

[tjp13073-bib-0007] Berger SL & Sassone‐Corsi P (2016). Metabolic signaling to chromatin. Cold Spring Harb Perspect Biol 8, a019463. 26492570 10.1101/cshperspect.a019463 PMC5088527

[tjp13073-bib-0008] Bhagwat AS & Vakoc CR (2015). Targeting transcription factors in cancer. Trends Cancer 1, 53–65. 26645049 10.1016/j.trecan.2015.07.001 PMC4669894

[tjp13073-bib-0009] Blevins MA , Huang M & Zhao R (2017). The role of CtBP1 in oncogenic processes and its potential as a therapeutic target. Mol Cancer Ther 16, 981–990. 28576945 10.1158/1535-7163.MCT-16-0592 PMC5458631

[tjp13073-bib-0010] Bourgeois A , Lambert C , Habbout K , Ranchoux B , Paquet‐Marceau S , Trinh I , Breuils‐Bonnet S , Paradis R , Nadeau V , Paulin R , Provencher S , Bonnet S & Boucherat O (2018). FOXM1 promotes pulmonary artery smooth muscle cell expansion in pulmonary arterial hypertension. J Mol Med (Berl) 96, 223–235. 29290032 10.1007/s00109-017-1619-0

[tjp13073-bib-0011] Bradner JE , Hnisz D & Young RA (2017). Transcriptional addiction in cancer. Cell 168, 629–643. 28187285 10.1016/j.cell.2016.12.013 PMC5308559

[tjp13073-bib-0012] Brown JD , Lin CY , Duan Q , Griffin G , Federation AJ , Paranal RM , Bair S , Newton G , Lichtman AH , Kung AL , Yang T , Wang H , Luscinskas FW , Croce KJ , Bradner JE & Plutzky J (2014). NF‐κB directs dynamic super enhancer formation in inflammation and atherogenesis. Mol Cell 56, 219–231. 25263595 10.1016/j.molcel.2014.08.024 PMC4224636

[tjp13073-bib-0013] Brusselmans K , Compernolle V , Tjwa M , Wiesener MS , Maxwell PH , Collen D & Carmeliet P (2003). Heterozygous deficiency of hypoxia‐inducible factor‐2α protects mice against pulmonary hypertension and right ventricular dysfunction during prolonged hypoxia. J Clin Invest 111, 1519–1527. 12750401 10.1172/JCI15496 PMC155039

[tjp13073-bib-0014] Bryant AJ , Carrick RP , McConaha ME , Jones BR , Shay SD , Moore CS , Blackwell TR , Gladson S , Penner NL , Burman A , Tanjore H , Hemnes AR , Karwandyar AK , Polosukhin VV , Talati MA , Dong HJ , Gleaves LA , Carrier EJ , Gaskill C , Scott EW , Majka SM , Fessel JP , Haase VH , West JD , Blackwell TS & Lawson WE (2016). Endothelial HIF signaling regulates pulmonary fibrosis‐associated pulmonary hypertension. Am J Physiol Lung Cell Mol Physiol 310, L249–L262. 26637636 10.1152/ajplung.00258.2015 PMC4838140

[tjp13073-bib-0015] Budas GR , Boehm M , Kojonazarov B , Viswanathan G , Tian X , Veeroju S , Novoyatleva T , Grimminger F , Hinojosa‐Kirschenbaum F , Ghofrani HA , Weissmann N , Seeger W , Liles JT & Schermuly RT (2018). ASK1 inhibition halts disease progression in preclinical models of pulmonary arterial hypertension. Am J Respir Crit Care Med 197, 373–385. 28910144 10.1164/rccm.201703-0502OC

[tjp13073-bib-0016] Byun JS & Gardner K (2013). C‐terminal binding protein: a molecular link between metabolic imbalance and epigenetic regulation in breast cancer. Int J Cell Biol 2013, 647975. 23762064 10.1155/2013/647975 PMC3671672

[tjp13073-bib-0017] Cai SF , Chen CW & Armstrong SA (2015). Drugging chromatin in cancer: recent advances and novel approaches. Mol Cell 60, 561–570. 26590715 10.1016/j.molcel.2015.10.042 PMC4701197

[tjp13073-bib-0018] Caruso P , Dunmore BJ , Schlosser K , Schoors S , Dos Santos C , Perez‐Iratxeta C , Lavoie JR , Zhang H , Long L , Flockton AR , Frid MG , Upton PD , D'Alessandro A , Hadinnapola C , Kiskin FN , Taha M , Hurst LA , Ormiston ML , Hata A , Stenmark KR , Carmeliet P , Stewart DJ & Morrell NW (2017). Identification of microRNA‐124 as a major regulator of enhanced endothelial cell glycolysis in pulmonary arterial hypertension via PTBP1 (polypyrimidine tract binding protein) and pyruvate kinase M2. Circulation 136, 2451–2467. 28971999 10.1161/CIRCULATIONAHA.117.028034 PMC5736425

[tjp13073-bib-0019] Cavasin MA , Demos‐Davies K , Horn TR , Walker LA , Lemon DD , Birdsey N , Weiser‐Evans MC , Harral J , Irwin DC , Anwar A , Yeager ME , Li M , Watson PA , Nemenoff RA , Buttrick PM , Stenmark KR & McKinsey TA (2012). Selective class I histone deacetylase inhibition suppresses hypoxia‐induced cardiopulmonary remodeling through an antiproliferative mechanism. Circ Res 110, 739–748. 22282194 10.1161/CIRCRESAHA.111.258426 PMC3682822

[tjp13073-bib-0020] Choudhary C , Kumar C , Gnad F , Nielsen ML , Rehman M , Walther TC , Olsen JV & Mann M (2009). Lysine acetylation targets protein complexes and co‐regulates major cellular functions. Science 325, 834–840. 19608861 10.1126/science.1175371

[tjp13073-bib-0021] Church AC , Martin DH , Wadsworth R , Bryson G , Fisher AJ , Welsh DJ & Peacock AJ (2015). The reversal of pulmonary vascular remodeling through inhibition of p38 MAPK‐alpha: a potential novel anti‐inflammatory strategy in pulmonary hypertension. Am J Physiol Lung Cell Mol Physiol 309, L333–L347. 26024891 10.1152/ajplung.00038.2015 PMC4538235

[tjp13073-bib-0022] Cosma MP (2002). Ordered recruitment: gene‐specific mechanism of transcription activation. Mol Cell 10, 227–236. 12191469 10.1016/s1097-2765(02)00604-4

[tjp13073-bib-0023] Cowburn AS , Crosby A , Macias D , Branco C , Colaco RD , Southwood M , Toshner M , Crotty Alexander LE , Morrell NW , Chilvers ER & Johnson RS (2016). HIF2alpha‐arginase axis is essential for the development of pulmonary hypertension. Proc Natl Acad Sci U S A 113, 8801–8806. 27432976 10.1073/pnas.1602978113 PMC4978263

[tjp13073-bib-0024] Dai Z , Li M , Wharton J , Zhu MM & Zhao YY (2016). Prolyl‐4 hydroxylase 2 (PHD2) deficiency in endothelial cells and hematopoietic cells induces obliterative vascular remodeling and severe pulmonary arterial hypertension in mice and humans through hypoxia‐inducible factor‐2α. Circulation 133, 2447–2458. 27143681 10.1161/CIRCULATIONAHA.116.021494 PMC4907810

[tjp13073-bib-0025] Dai Z , Zhu MM , Peng Y , Jin H , Machireddy N , Qian Z , Zhang X & Zhao YY (2018). Endothelial and smooth muscle cell interaction via FoxM1 signaling mediates vascular remodeling and pulmonary hypertension. Am J Respir Crit Care Med (in press; 10.1164/rccm.201709-1835OC). PMC6222462 29664678

[tjp13073-bib-0026] D'Alessandro A , El Kasmi KC , Plecita‐Hlavata L , Jezek P , Li M , Zhang H , Gupte SA & Stenmark KR (2018). Hallmarks of pulmonary hypertension: mesenchymal and inflammatory cell metabolic reprogramming. Antioxid Redox Signal 28, 230–250. 28637353 10.1089/ars.2017.7217 PMC5737722

[tjp13073-bib-0027] Dayton TL , Jacks T & Vander Heiden MG (2016). PKM2, cancer metabolism, and the road ahead. EMBO Rep 17, 1721–1730. 27856534 10.15252/embr.201643300 PMC5283597

[tjp13073-bib-0028] De Raaf MA , Hussaini AA , Gomez‐Arroyo J , Kraskaukas D , Farkas D , Happe C , Voelkel NF & Bogaard HJ (2014). Histone deacetylase inhibition with trichostatin A does not reverse severe angioproliferative pulmonary hypertension in rats (2013 Grover Conference series). Pulm Circ 4, 237–243. 25006442 10.1086/675986 PMC4070779

[tjp13073-bib-0029] Dong G , Mao Q , Xia W , Xu Y , Wang J , Xu L & Jiang F (2016). PKM2 and cancer: The function of PKM2 beyond glycolysis. Oncol Lett 11, 1980–1986. 26998110 10.3892/ol.2016.4168 PMC4774429

[tjp13073-bib-0030] Duluc L , Ahmetaj‐Shala B , Mitchell J , Abdul‐Salam VB , Mahomed AS , Aldabbous L , Oliver E , Iannone L , Dubois OD , Storck EM , Tate EW , Zhao L , Wilkins MR & Wojciak‐Stothard B (2017). Tipifarnib prevents development of hypoxia‐induced pulmonary hypertension. Cardiovasc Res 113, 276–287. 28395021 10.1093/cvr/cvw258 PMC5408956

[tjp13073-bib-0031] Farkas D , Alhussaini AA , Kraskauskas D , Kraskauskiene V , Cool CD , Nicolls MR , Natarajan R & Farkas L (2014). Nuclear factor κB inhibition reduces lung vascular lumen obliteration in severe pulmonary hypertension in rats. Am J Respir Cell Mol Biol 51, 413–425. 24684441 10.1165/rcmb.2013-0355OC PMC4189489

[tjp13073-bib-0032] Filippakopoulos P , Picaud S , Fedorov O , Keller M , Wrobel M , Morgenstern O , Bracher F & Knapp S (2012). Benzodiazepines and benzotriazepines as protein interaction inhibitors targeting bromodomains of the BET family. Bioorg Med Chem 20, 1878–1886. 22137933 10.1016/j.bmc.2011.10.080 PMC4748212

[tjp13073-bib-0033] Frid MG , Brunetti JA , Burke DL , Carpenter TC , Davie NJ , Reeves JT , Roedersheimer MT , van Rooijen N & Stenmark KR (2006). Hypoxia‐induced pulmonary vascular remodeling requires recruitment of circulating mesenchymal precursors of a monocyte/macrophage lineage. Am J Pathol 168, 659–669. 16436679 10.2353/ajpath.2006.050599 PMC1606508

[tjp13073-bib-0034] Gambaryan N , Perros F , Montani D , Cohen‐Kaminsky S , Mazmanian GM & Humbert M (2010). Imatinib inhibits bone marrow‐derived c‐kit^+^ cell mobilisation in hypoxic pulmonary hypertension. Eur Respir J 36, 1209–1211. 21037371 10.1183/09031936.00052210

[tjp13073-bib-0035] Garat CV , Crossno JT Jr , Sullivan TM , Reusch JE & Klemm DJ (2013). Inhibition of phosphatidylinositol 3‐kinase/Akt signaling attenuates hypoxia‐induced pulmonary artery remodeling and suppresses CREB depletion in arterial smooth muscle cells. J Cardiovasc Pharmacol 62, 539–548. 24084215 10.1097/FJC.0000000000000014 PMC4143163

[tjp13073-bib-0036] Ghataorhe P , Rhodes CJ , Harbaum L , Attard M , Wharton J & Wilkins MR (2017). Pulmonary arterial hypertension – progress in understanding the disease and prioritizing strategies for drug development. J Intern Med 282, 129–141. 28524624 10.1111/joim.12623

[tjp13073-bib-0037] Grivennikov SI & Karin M (2010). Dangerous liaisons: STAT3 and NF‐κB collaboration and crosstalk in cancer. Cytokine Growth Factor Rev 21, 11–19. 20018552 10.1016/j.cytogfr.2009.11.005 PMC2834864

[tjp13073-bib-0038] Guignabert C , Bailly S & Humbert M (2017). Restoring BMPRII functions in pulmonary arterial hypertension: opportunities, challenges and limitations. Expert Opin Ther Targets 21, 181–190. 28001443 10.1080/14728222.2017.1275567

[tjp13073-bib-0039] Guzy RD , Mack MM & Schumacker PT (2007). Mitochondrial complex III is required for hypoxia‐induced ROS production and gene transcription in yeast. Antioxid Redox Signal 9, 1317–1328. 17627464 10.1089/ars.2007.1708

[tjp13073-bib-0040] Halliday GM , Bock VL , Moloney FJ & Lyons JG (2009). SWI/SNF: a chromatin‐remodelling complex with a role in carcinogenesis. Int J Biochem Cell Biol 41, 725–728. 18723114 10.1016/j.biocel.2008.04.026

[tjp13073-bib-0041] Hancock RL , Dunne K , Walport LJ , Flashman E & Kawamura A (2015). Epigenetic regulation by histone demethylases in hypoxia. Epigenomics 7, 791–811. 25832587 10.2217/epi.15.24

[tjp13073-bib-0042] Hassoun PM , Mouthon L , Barbera JA , Eddahibi S , Flores SC , Grimminger F , Jones PL , Maitland ML , Michelakis ED , Morrell NW , Newman JH , Rabinovitch M , Schermuly R , Stenmark KR , Voelkel NF , Yuan JX & Humbert M (2009). Inflammation, growth factors, and pulmonary vascular remodeling. J Am Coll Cardiol 54, S10–19. 19555853 10.1016/j.jacc.2009.04.006

[tjp13073-bib-0043] Hayashida K , Fujita J , Miyake Y , Kawada H , Ando K , Ogawa S & Fukuda K (2005). Bone marrow‐derived cells contribute to pulmonary vascular remodeling in hypoxia‐induced pulmonary hypertension. Chest 127, 1793–1798. 15888860 10.1378/chest.127.5.1793

[tjp13073-bib-0044] Heerboth S , Lapinska K , Snyder N , Leary M , Rollinson S & Sarkar S (2014). Use of epigenetic drugs in disease: an overview. Genet Epigenet 6, 9–19. 25512710 10.4137/GEG.S12270 PMC4251063

[tjp13073-bib-0045] Heinz S , Romanoski CE , Benner C & Glass CK (2015). The selection and function of cell type‐specific enhancers. Nat Rev Mol Cell Biol 16, 144–154. 25650801 10.1038/nrm3949 PMC4517609

[tjp13073-bib-0046] Hickey MM , Richardson T , Wang T , Mosqueira M , Arguiri E , Yu H , Yu QC , Solomides CC , Morrisey EE , Khurana TS , Christofidou‐Solomidou M & Simon MC (2010). The von Hippel‐Lindau Chuvash mutation promotes pulmonary hypertension and fibrosis in mice. J Clin Invest 120, 827–839. 20197624 10.1172/JCI36362 PMC2827942

[tjp13073-bib-0047] Hnisz D , Abraham BJ , Lee TI , Lau A , Saint‐Andre V , Sigova AA , Hoke HA & Young RA (2013). Super‐enhancers in the control of cell identity and disease. Cell 155, 934–947. 24119843 10.1016/j.cell.2013.09.053 PMC3841062

[tjp13073-bib-0048] Hosokawa S , Haraguchi G , Sasaki A , Arai H , Muto S , Itai A , Doi S , Mizutani S & Isobe M (2013). Pathophysiological roles of nuclear factor kappaB (NF‐κB) in pulmonary arterial hypertension: effects of synthetic selective NF‐κB inhibitor IMD‐0354. Cardiovasc Res 99, 35–43. 23631839 10.1093/cvr/cvt105

[tjp13073-bib-0049] Huang J , Kaminski PM , Edwards JG , Yeh A , Wolin MS , Frishman WH , Gewitz MH & Mathew R (2008). Pyrrolidine dithiocarbamate restores endothelial cell membrane integrity and attenuates monocrotaline‐induced pulmonary artery hypertension. Am J Physiol Lung Cell Mol Physiol 294, L1250–L1259. 18390833 10.1152/ajplung.00069.2007 PMC2441448

[tjp13073-bib-0050] Jacquin S , Rincheval V , Mignotte B , Richard S , Humbert M , Mercier O , Londono‐Vallejo A , Fadel E & Eddahibi S (2015). Inactivation of p53 is sufficient to induce development of pulmonary hypertension in rats. PLoS One 10, e0131940. 26121334 10.1371/journal.pone.0131940 PMC4488287

[tjp13073-bib-0051] Jang MK , Mochizuki K , Zhou M , Jeong HS , Brady JN & Ozato K (2005). The bromodomain protein Brd4 is a positive regulatory component of P‐TEFb and stimulates RNA polymerase II‐dependent transcription. Mol Cell 19, 523–534. 16109376 10.1016/j.molcel.2005.06.027

[tjp13073-bib-0052] Jones PA , Issa JP & Baylin S (2016). Targeting the cancer epigenome for therapy. Nat Rev Genet 17, 630–641. 27629931 10.1038/nrg.2016.93

[tjp13073-bib-0053] Kapitsinou PP , Rajendran G , Astleford L , Michael M , Schonfeld MP , Fields T , Shay S , French JL , West J & Haase VH (2016). The endothelial prolyl‐4‐hydroxylase domain 2/hypoxia‐inducible factor 2 axis regulates pulmonary artery pressure in mice. Mol Cell Biol 36, 1584–1594. 26976644 10.1128/MCB.01055-15 PMC4859687

[tjp13073-bib-0054] Kimura S , Egashira K , Chen L , Nakano K , Iwata E , Miyagawa M , Tsujimoto H , Hara K , Morishita R , Sueishi K , Tominaga R & Sunagawa K (2009). Nanoparticle‐mediated delivery of nuclear factor κB decoy into lungs ameliorates monocrotaline‐induced pulmonary arterial hypertension. Hypertension 53, 877–883. 19307469 10.1161/HYPERTENSIONAHA.108.121418

[tjp13073-bib-0055] Kornberg RD (2001). The eukaryotic gene transcription machinery. Biol Chem 382, 1103–1107. 11592390 10.1515/BC.2001.140

[tjp13073-bib-0056] Koschmann C , Nunez FJ , Mendez F , Brosnan‐Cashman JA , Meeker AK , Lowenstein PR & Castro MG (2017). Mutated chromatin regulatory factors as tumor drivers in cancer. Cancer Res 77, 227–233. 28062403 10.1158/0008-5472.CAN-16-2301 PMC5243833

[tjp13073-bib-0057] Kreuz S & Fischle W (2016). Oxidative stress signaling to chromatin in health and disease. Epigenomics 8, 843–862. 27319358 10.2217/epi-2016-0002 PMC5619053

[tjp13073-bib-0058] Kuppuswamy M , Vijayalingam S , Zhao LJ , Zhou Y , Subramanian T , Ryerse J & Chinnadurai G (2008). Role of the PLDLS‐binding cleft region of CtBP1 in recruitment of core and auxiliary components of the corepressor complex. Mol Cell Biol 28, 269–281. 17967884 10.1128/MCB.01077-07 PMC2223311

[tjp13073-bib-0059] Lane KB , Blackwell TR , Runo J , Wheeler L , Phillips JA 3rd & Loyd JE (2005). Aberrant signal transduction in pulmonary hypertension. Chest 128, 564S–565S. 10.1378/chest.128.6_suppl.564S-a 16373826

[tjp13073-bib-0060] Lau EMT , Giannoulatou E , Celermajer DS & Humbert M (2017). Epidemiology and treatment of pulmonary arterial hypertension. Nat Rev Cardiol 14, 603–614. 28593996 10.1038/nrcardio.2017.84

[tjp13073-bib-0061] Lavin Y , Winter D , Blecher‐Gonen R , David E , Keren‐Shaul H , Merad M , Jung S & Amit I (2014). Tissue‐resident macrophage enhancer landscapes are shaped by the local microenvironment. Cell 159, 1312–1326. 25480296 10.1016/j.cell.2014.11.018 PMC4437213

[tjp13073-bib-0062] Lee TI & Young RA (2013). Transcriptional regulation and its misregulation in disease. Cell 152, 1237–1251. 23498934 10.1016/j.cell.2013.02.014 PMC3640494

[tjp13073-bib-0063] Li L , Wei C , Kim IK , Janssen‐Heininger Y & Gupta S (2014). Inhibition of nuclear factor‐κB in the lungs prevents monocrotaline‐induced pulmonary hypertension in mice. Hypertension 63, 1260–1269. 24614212 10.1161/HYPERTENSIONAHA.114.03220

[tjp13073-bib-0064] Li M , Riddle S , Zhang H , D'Alessandro A , Flockton A , Serkova NJ , Hansen KC , Moldvan R , McKeon BA , Frid M , Kumar S , Li H , Liu H , Caanovas A , Medrano JF , Thomas MG , Iloska D , Plecita‐Hlavata L , Jezek P , Pullamsetti S , Fini MA , El Kasmi KC , Zhang Q & Stenmark KR (2016). Metabolic reprogramming regulates the proliferative and inflammatory phenotype of adventitial fibroblasts in pulmonary hypertension through the transcriptional corepressor C‐terminal binding protein‐1. Circulation 134, 1105–1121. 27562971 10.1161/CIRCULATIONAHA.116.023171 PMC5069179

[tjp13073-bib-0065] Li M , Riddle SR , Frid MG , El Kasmi KC , McKinsey TA , Sokol RJ , Strassheim D , Meyrick B , Yeager ME , Flockton AR , McKeon BA , Lemon DD , Horn TR , Anwar A , Barajas C & Stenmark KR (2011). Emergence of fibroblasts with a proinflammatory epigenetically altered phenotype in severe hypoxic pulmonary hypertension. J Immunol 187, 2711–2722. 21813768 10.4049/jimmunol.1100479 PMC3159707

[tjp13073-bib-0066] Luo W , Hu H , Chang R , Zhong J , Knabel M , O'Meally R , Cole RN , Pandey A & Semenza GL (2011). Pyruvate kinase M2 is a PHD3‐stimulated coactivator for hypoxia‐inducible factor 1. Cell 145, 732–744. 21620138 10.1016/j.cell.2011.03.054 PMC3130564

[tjp13073-bib-0067] Luo W & Semenza GL (2011). Pyruvate kinase M2 regulates glucose metabolism by functioning as a coactivator for hypoxia‐inducible factor 1 in cancer cells. Oncotarget 2, 551–556. 21709315 10.18632/oncotarget.299 PMC3248177

[tjp13073-bib-0068] Lythgoe MP , Rhodes CJ , Ghataorhe P , Attard M , Wharton J & Wilkins MR (2016). Why drugs fail in clinical trials in pulmonary arterial hypertension, and strategies to succeed in the future. Pharmacol Ther 164, 195–203. 27133570 10.1016/j.pharmthera.2016.04.012

[tjp13073-bib-0069] McGrath J & Trojer P (2015). Targeting histone lysine methylation in cancer. Pharmacol Ther 150, 1–22. 25578037 10.1016/j.pharmthera.2015.01.002

[tjp13073-bib-0070] Maron BA & Galie N (2016). Diagnosis, treatment, and clinical management of pulmonary arterial hypertension in the contemporary era: a review. JAMA Cardiol 1, 1056–1065. 27851839 10.1001/jamacardio.2016.4471 PMC5177491

[tjp13073-bib-0071] Meloche J , Lampron MC , Nadeau V , Maltais M , Potus F , Lambert C , Tremblay E , Vitry G , Breuils‐Bonnet S , Boucherat O , Charbonneau E , Provencher S , Paulin R & Bonnet S (2017). Implication of inflammation and epigenetic readers in coronary artery remodeling in patients with pulmonary arterial hypertension. Arterioscler Thromb Vasc Biol 37, 1513–1523. 28473439 10.1161/ATVBAHA.117.309156

[tjp13073-bib-0072] Meloche J , Potus F , Vaillancourt M , Bourgeois A , Johnson I , Deschamps L , Chabot S , Ruffenach G , Henry S , Breuils‐Bonnet S , Tremblay E , Nadeau V , Lambert C , Paradis R , Provencher S & Bonnet S (2015). Bromodomain‐containing protein 4: the epigenetic origin of pulmonary arterial hypertension. Circ Res 117, 525–535. 26224795 10.1161/CIRCRESAHA.115.307004

[tjp13073-bib-0073] Merklinger SL , Jones PL , Martinez EC & Rabinovitch M (2005). Epidermal growth factor receptor blockade mediates smooth muscle cell apoptosis and improves survival in rats with pulmonary hypertension. Circulation 112, 423–431. 16027270 10.1161/CIRCULATIONAHA.105.540542

[tjp13073-bib-0074] Mizuno S , Bogaard HJ , Kraskauskas D , Alhussaini A , Gomez‐Arroyo J , Voelkel NF & Ishizaki T (2011). p53 gene deficiency promotes hypoxia‐induced pulmonary hypertension and vascular remodeling in mice. Am J Physiol Lung Cell Mol Physiol 300, L753–L761. 21335523 10.1152/ajplung.00286.2010

[tjp13073-bib-0075] Morecroft I , Doyle B , Nilsen M , Kolch W , Mair K & Maclean MR (2011). Mice lacking the Raf‐1 kinase inhibitor protein exhibit exaggerated hypoxia‐induced pulmonary hypertension. Br J Pharmacol 163, 948–963. 21385176 10.1111/j.1476-5381.2011.01305.x PMC3130942

[tjp13073-bib-0076] Morecroft I , Pang L , Baranowska M , Nilsen M , Loughlin L , Dempsie Y , Millet C & MacLean MR (2010). In vivo effects of a combined 5‐HT1B receptor/SERT antagonist in experimental pulmonary hypertension. Cardiovasc Res 85, 593–603. 19736308 10.1093/cvr/cvp306

[tjp13073-bib-0077] Morgan MA & Shilatifard A (2015). Chromatin signatures of cancer. Genes Dev 29, 238–249. 25644600 10.1101/gad.255182.114 PMC4318141

[tjp13073-bib-0078] Morrell ED , Tsai BM , Crisostomo PR , Hammoud ZT & Meldrum DR (2006). Experimental therapies for hypoxia‐induced pulmonary hypertension during acute lung injury. Shock 25, 214–226. 16552352 10.1097/01.shk.0000191380.44972.46

[tjp13073-bib-0079] Mortimer HJ , Peacock AJ , Kirk A & Welsh DJ (2007). p38 MAP kinase: essential role in hypoxia‐mediated human pulmonary artery fibroblast proliferation. Pulm Pharmacol Ther 20, 718–725. 17055760 10.1016/j.pupt.2006.08.007

[tjp13073-bib-0080] Murakami A , Wang L , Kalhorn S , Schraml P , Rathmell WK , Tan AC , Nemenoff R , Stenmark K , Jiang BH , Reyland ME , Heasley L & Hu CJ (2017). Context‐dependent role for chromatin remodeling component PBRM1/BAF180 in clear cell renal cell carcinoma. Oncogenesis 6, e287. 28092369 10.1038/oncsis.2016.89 PMC5294252

[tjp13073-bib-0081] Nemenoff RA , Simpson PA , Furgeson SB , Kaplan‐Albuquerque N , Crossno J , Garl PJ , Cooper J & Weiser‐Evans MC (2008). Targeted deletion of PTEN in smooth muscle cells results in vascular remodeling and recruitment of progenitor cells through induction of stromal cell‐derived factor‐1α. Circ Res 102, 1036–1045. 18340011 10.1161/CIRCRESAHA.107.169896

[tjp13073-bib-0082] Nozik‐Grayck E , Suliman HB , Majka S , Albietz J , Van Rheen Z , Roush K & Stenmark KR (2008). Lung EC‐SOD overexpression attenuates hypoxic induction of Egr‐1 and chronic hypoxic pulmonary vascular remodeling. Am J Physiol Lung Cell Mol Physiol 295, L422–L430. 18599502 10.1152/ajplung.90293.2008 PMC2536799

[tjp13073-bib-0083] Nozik‐Grayck E , Woods C , Taylor JM , Benninger RK , Johnson RD , Villegas LR , Stenmark KR , Harrison DG , Majka SM , Irwin D & Farrow KN (2014). Selective depletion of vascular EC‐SOD augments chronic hypoxic pulmonary hypertension. Am J Physiol Lung Cell Mol Physiol 307, L868–L876. 25326578 10.1152/ajplung.00096.2014 PMC4254965

[tjp13073-bib-0084] Orriols M , Gomez‐Puerto MC & Ten Dijke P (2017). BMP type II receptor as a therapeutic target in pulmonary arterial hypertension. Cell Mol Life Sci 74, 2979–2995. 28447104 10.1007/s00018-017-2510-4 PMC5501910

[tjp13073-bib-0085] Paddenberg R , Ishaq B , Goldenberg A , Faulhammer P , Rose F , Weissmann N , Braun‐Dullaeus RC & Kummer W (2003). Essential role of complex II of the respiratory chain in hypoxia‐induced ROS generation in the pulmonary vasculature. Am J Physiol Lung Cell Mol Physiol 284, L710–L719. 12676762 10.1152/ajplung.00149.2002

[tjp13073-bib-0086] Paulin R , Courboulin A , Meloche J , Mainguy V , Dumas de la Roque E , Saksouk N , Cote J , Provencher S , Sussman MA & Bonnet S (2011a). Signal transducers and activators of transcription‐3/pim1 axis plays a critical role in the pathogenesis of human pulmonary arterial hypertension. Circulation 123, 1205–1215. 21382889 10.1161/CIRCULATIONAHA.110.963314 PMC3545712

[tjp13073-bib-0087] Paulin R , Meloche J & Bonnet S (2012). STAT3 signaling in pulmonary arterial hypertension. JAKSTAT 1, 223–233. 24058777 10.4161/jkst.22366 PMC3670278

[tjp13073-bib-0088] Paulin R , Meloche J , Jacob MH , Bisserier M , Courboulin A & Bonnet S (2011b). Dehydroepiandrosterone inhibits the Src/STAT3 constitutive activation in pulmonary arterial hypertension. Am J Physiol Heart Circ Physiol 301, H1798–H1809. 21890685 10.1152/ajpheart.00654.2011

[tjp13073-bib-0089] Pawlus MR & Hu CJ (2013). Enhanceosomes as integrators of hypoxia inducible factor (HIF) and other transcription factors in the hypoxic transcriptional response. Cell Signal 25, 1895–1903. 23707522 10.1016/j.cellsig.2013.05.018 PMC3700616

[tjp13073-bib-0090] Plecita‐Hlavata L , D'Alessandro A , El Kasmi K , Li M , Zhang H , Jezek P & Stenmark KR (2017). Metabolic reprogramming and redox signaling in pulmonary hypertension. Adv Exp Med Biol 967, 241–260. 29047090 10.1007/978-3-319-63245-2_14

[tjp13073-bib-0091] Plecita‐Hlavata L , Tauber J , Li M , Zhang H , Flockton AR , Pullamsetti SS , Chelladurai P , D'Alessandro A , El Kasmi KC , Jezek P & Stenmark KR (2016). Constitutive reprogramming of fibroblast mitochondrial metabolism in pulmonary hypertension. Am J Respir Cell Mol Biol 55, 47–57. 26699943 10.1165/rcmb.2015-0142OC PMC4942204

[tjp13073-bib-0092] Price LC , Caramori G , Perros F , Meng C , Gambaryan N , Dorfmuller P , Montani D , Casolari P , Zhu J , Dimopoulos K , Shao D , Girerd B , Mumby S , Proudfoot A , Griffiths M , Papi A , Humbert M , Adcock IM & Wort SJ (2013). Nuclear factor κ‐B is activated in the pulmonary vessels of patients with end‐stage idiopathic pulmonary arterial hypertension. PLoS One 8, e75415. 24124488 10.1371/journal.pone.0075415 PMC3790752

[tjp13073-bib-0093] Prickaerts P , Adriaens ME , Beucken TVD , Koch E , Dubois L , Dahlmans VEH , Gits C , Evelo CTA , Chan‐Seng‐Yue M , Wouters BG & Voncken JW (2016). Hypoxia increases genome‐wide bivalent epigenetic marking by specific gain of H3K27me3. Epigenetics Chromatin 9, 46. 27800026 10.1186/s13072-016-0086-0 PMC5080723

[tjp13073-bib-0094] Probst AV , Dunleavy E & Almouzni G (2009). Epigenetic inheritance during the cell cycle. Nat Rev Mol Cell Biol 10, 192–206. 19234478 10.1038/nrm2640

[tjp13073-bib-0095] Pugliese SC , Poth JM , Fini MA , Olschewski A , El Kasmi KC & Stenmark KR (2015). The role of inflammation in hypoxic pulmonary hypertension: from cellular mechanisms to clinical phenotypes. Am J Physiol Lung Cell Mol Physiol 308, L229–L252. 25416383 10.1152/ajplung.00238.2014 PMC4338929

[tjp13073-bib-0096] Pullamsetti SS , Berghausen EM , Dabral S , Tretyn A , Butrous E , Savai R , Butrous G , Dahal BK , Brandes RP , Ghofrani HA , Weissmann N , Grimminger F , Seeger W , Rosenkranz S & Schermuly RT (2012). Role of Src tyrosine kinases in experimental pulmonary hypertension. Arterioscler Thromb Vasc Biol 32, 1354–1365. 22516066 10.1161/ATVBAHA.112.248500

[tjp13073-bib-0097] Pullamsetti SS , Perros F , Chelladurai P , Yuan J & Stenmark K (2016). Transcription factors, transcriptional coregulators, and epigenetic modulation in the control of pulmonary vascular cell phenotype: therapeutic implications for pulmonary hypertension (2015 Grover Conference series). Pulm Circ 6, 448–464. 28090287 10.1086/688908 PMC5210074

[tjp13073-bib-0098] Pullamsetti SS , Savai R , Seeger W & Goncharova EA (2017). Translational advances in the field of pulmonary hypertension. from cancer biology to new pulmonary arterial hypertension therapeutics. targeting cell growth and proliferation signaling hubs. Am J Respir Crit Care Med 195, 425–437. 27627135 10.1164/rccm.201606-1226PP PMC5803657

[tjp13073-bib-0099] Rabinovitch M , Guignabert C , Humbert M & Nicolls MR (2014). Inflammation and immunity in the pathogenesis of pulmonary arterial hypertension. Circ Res 115, 165–175. 24951765 10.1161/CIRCRESAHA.113.301141 PMC4097142

[tjp13073-bib-0100] Ramiro‐Diaz JM , Nitta CH , Maston LD , Codianni S , Giermakowska W , Resta TC & Gonzalez Bosc LV (2013). NFAT is required for spontaneous pulmonary hypertension in superoxide dismutase 1 knockout mice. Am J Physiol Lung Cell Mol Physiol 304, L613–L625. 23475768 10.1152/ajplung.00408.2012 PMC3652021

[tjp13073-bib-0101] Reid MA , Dai Z & Locasale JW (2017). The impact of cellular metabolism on chromatin dynamics and epigenetics. Nat Cell Biol 19, 1298–1306. 29058720 10.1038/ncb3629 PMC5886854

[tjp13073-bib-0102] Reisman D , Glaros S & Thompson EA (2009). The SWI/SNF complex and cancer. Oncogene 28, 1653–1668. 19234488 10.1038/onc.2009.4

[tjp13073-bib-0103] Ries D & Meisterernst M (2011). Control of gene transcription by Mediator in chromatin. Semin Cell Dev Biol 22, 735–740. 21864698 10.1016/j.semcdb.2011.08.004

[tjp13073-bib-0104] Roe JS , Mercan F , Rivera K , Pappin DJ & Vakoc CR (2015). BET bromodomain inhibition suppresses the function of hematopoietic transcription factors in acute myeloid leukemia. Mol Cell 58, 1028–1039. 25982114 10.1016/j.molcel.2015.04.011 PMC4475489

[tjp13073-bib-0105] Said SI , Hamidi SA , Dickman KG , Szema AM , Lyubsky S , Lin RZ , Jiang YP , Chen JJ , Waschek JA & Kort S (2007). Moderate pulmonary arterial hypertension in male mice lacking the vasoactive intestinal peptide gene. Circulation 115, 1260–1268. 17309917 10.1161/CIRCULATIONAHA.106.681718

[tjp13073-bib-0106] Savai R , Al‐Tamari HM , Sedding D , Kojonazarov B , Muecke C , Teske R , Capecchi MR , Weissmann N , Grimminger F , Seeger W , Schermuly RT & Pullamsetti SS (2014). Pro‐proliferative and inflammatory signaling converge on FoxO1 transcription factor in pulmonary hypertension. Nat Med 20, 1289–1300. 25344740 10.1038/nm.3695

[tjp13073-bib-0107] Sawada H , Mitani Y , Maruyama J , Jiang BH , Ikeyama Y , Dida FA , Yamamoto H , Imanaka‐Yoshida K , Shimpo H , Mizoguchi A , Maruyama K & Komada Y (2007). A nuclear factor‐κB inhibitor pyrrolidine dithiocarbamate ameliorates pulmonary hypertension in rats. Chest 132, 1265–1274. 17934115 10.1378/chest.06-2243

[tjp13073-bib-0108] Schermuly RT , Dony E , Ghofrani HA , Pullamsetti S , Savai R , Roth M , Sydykov A , Lai YJ , Weissmann N , Seeger W & Grimminger F (2005). Reversal of experimental pulmonary hypertension by PDGF inhibition. J Clin Invest 115, 2811–2821. 16200212 10.1172/JCI24838 PMC1236676

[tjp13073-bib-0109] Schermuly RT , Ghofrani HA , Wilkins MR & Grimminger F (2011). Mechanisms of disease: pulmonary arterial hypertension. Nat Rev Cardiol 8, 443–455. 21691314 10.1038/nrcardio.2011.87 PMC7097518

[tjp13073-bib-0110] Schonenberger D , Harlander S , Rajski M , Jacobs RA , Lundby AK , Adlesic M , Hejhal T , Wild PJ , Lundby C & Frew IJ (2016). Formation of renal cysts and tumors in Vhl/Trp53‐deficient mice requires HIF‐1α and HIF‐2α. Cancer Res 76, 2025–2036. 26759234 10.1158/0008-5472.CAN-15-1859

[tjp13073-bib-0111] Shen C , Beroukhim R , Schumacher SE , Zhou J , Chang M , Signoretti S & Kaelin WG Jr (2011). Genetic and functional studies implicate HIF1α as a 14q kidney cancer suppressor gene. Cancer Discov 1, 222–235. 22037472 10.1158/2159-8290.CD-11-0098 PMC3202343

[tjp13073-bib-0112] Shlyueva D , Stampfel G & Stark A (2014). Transcriptional enhancers: from properties to genome‐wide predictions. Nat Rev Genet 15, 272–286. 24614317 10.1038/nrg3682

[tjp13073-bib-0113] Simonneau G , Gatzoulis MA , Adatia I , Celermajer D , Denton C , Ghofrani A , Gomez Sanchez MA , Krishna Kumar R , Landzberg M , Machado RF , Olschewski H , Robbins IM & Souza R (2013). Updated clinical classification of pulmonary hypertension. J Am Coll Cardiol 62, D34–41. 24355639 10.1016/j.jacc.2013.10.029

[tjp13073-bib-0114] Singh A , Greninger P , Rhodes D , Koopman L , Violette S , Bardeesy N & Settleman J (2009). A gene expression signature associated with “K‐Ras addiction” reveals regulators of EMT and tumor cell survival. Cancer Cell 15, 489–500. 19477428 10.1016/j.ccr.2009.03.022 PMC2743093

[tjp13073-bib-0115] Slingerland M , Guchelaar HJ & Gelderblom H (2014). Histone deacetylase inhibitors: an overview of the clinical studies in solid tumors. Anticancer Drugs 25, 140–149. 24185382 10.1097/CAD.0000000000000040

[tjp13073-bib-0116] Smith E & Shilatifard A (2014). Enhancer biology and enhanceropathies. Nat Struct Mol Biol 21, 210–219. 24599251 10.1038/nsmb.2784

[tjp13073-bib-0117] Spange S , Wagner T , Heinzel T & Kramer OH (2009). Acetylation of non‐histone proteins modulates cellular signalling at multiple levels. Int J Biochem Cell Biol 41, 185–198. 18804549 10.1016/j.biocel.2008.08.027

[tjp13073-bib-0118] Spitz F & Furlong EE (2012). Transcription factors: from enhancer binding to developmental control. Nat Rev Genet 13, 613–626. 22868264 10.1038/nrg3207

[tjp13073-bib-0119] Stenmark KR , Frid MG , Graham BB & Tuder RM (2018). Dynamic and diverse changes in the functional properties of vascular smooth muscle cells in pulmonary hypertension. Cardiovasc Res 114, 551–564. 29385432 10.1093/cvr/cvy004 PMC5852549

[tjp13073-bib-0120] Stenmark KR , Frid MG , Yeager M , Li M , Riddle S , McKinsey T & El Kasmi KC (2012). Targeting the adventitial microenvironment in pulmonary hypertension: A potential approach to therapy that considers epigenetic change. Pulm Circ 2, 3–14. 22558514 10.4103/2045-8932.94817 PMC3342746

[tjp13073-bib-0121] Stenmark KR , Meyrick B , Galie N , Mooi WJ & McMurtry IF (2009). Animal models of pulmonary arterial hypertension: the hope for etiological discovery and pharmacological cure. Am J Physiol Lung Cell Mol Physiol 297, L1013–L1032. 19748998 10.1152/ajplung.00217.2009

[tjp13073-bib-0122] Stenmark KR & Rabinovitch M (2010). Emerging therapies for the treatment of pulmonary hypertension. Pediatr Crit Care Med 11, S85–90. 20216170 10.1097/PCC.0b013e3181c76db3

[tjp13073-bib-0123] Stenmark KR , Tuder RM & El Kasmi KC (2015). Metabolic reprogramming and inflammation act in concert to control vascular remodeling in hypoxic pulmonary hypertension. J Appl Physiol (1985) 119, 1164–1172. 25930027 10.1152/japplphysiol.00283.2015 PMC4816410

[tjp13073-bib-0124] Sur I & Taipale J (2016). The role of enhancers in cancer. Nat Rev 16, 483–493. 10.1038/nrc.2016.62 27364481

[tjp13073-bib-0125] Sutendra G & Michelakis ED (2014). The metabolic basis of pulmonary arterial hypertension. Cell Metab 19, 558–573. 24508506 10.1016/j.cmet.2014.01.004

[tjp13073-bib-0126] Tan Q , Kerestes H , Percy MJ , Pietrofesa R , Chen L , Khurana TS , Christofidou‐Solomidou M , Lappin TR & Lee FS (2013). Erythrocytosis and pulmonary hypertension in a mouse model of human *HIF2A* gain of function mutation. J Biol Chem 288, 17134–17144. 23640890 10.1074/jbc.M112.444059 PMC3682519

[tjp13073-bib-0127] Tang H , Babicheva A , McDermott KM , Gu Y , Ayon RJ , Song S , Wang Z , Gupta A , Zhou T , Sun X , Dash S , Wang Z , Balistrieri A , Zheng Q , Cordery AG , Desai AA , Rischard F , Khalpey Z , Wang J , Black SM , Garcia JGN , Makino A & Yuan JX (2018). Endothelial HIF‐2α contributes to severe pulmonary hypertension due to endothelial‐to‐mesenchymal transition. Am J Physiol Lung Cell Mol Physiol 314, L256–L275. 29074488 10.1152/ajplung.00096.2017 PMC5866501

[tjp13073-bib-0128] Tang H , Chen J , Fraidenburg DR , Song S , Sysol JR , Drennan AR , Offermanns S , Ye RD , Bonini MG , Minshall RD , Garcia JG , Machado RF , Makino A & Yuan JX (2015). Deficiency of Akt1, but not Akt2, attenuates the development of pulmonary hypertension. Am J Physiol Lung Cell Mol Physiol 308, L208–L220. 25416384 10.1152/ajplung.00242.2014 PMC4338938

[tjp13073-bib-0129] Tian W , Jiang X , Tamosiuniene R , Sung YK , Qian J , Dhillon G , Gera L , Farkas L , Rabinovitch M , Zamanian RT , Inayathullah M , Fridlib M , Rajadas J , Peters‐Golden M , Voelkel NF & Nicolls MR (2013). Blocking macrophage leukotriene b4 prevents endothelial injury and reverses pulmonary hypertension. Sci Transl Med 5, 200ra117. 10.1126/scitranslmed.3006674 PMC4016764 23986401

[tjp13073-bib-0130] Tuder RM , Archer SL , Dorfmuller P , Erzurum SC , Guignabert C , Michelakis E , Rabinovitch M , Schermuly R , Stenmark KR & Morrell NW (2013). Relevant issues in the pathology and pathobiology of pulmonary hypertension. J Am Coll Cardiol 62, D4–12. 24355640 10.1016/j.jacc.2013.10.025 PMC3970402

[tjp13073-bib-0131] Voss TC & Hager GL (2014). Dynamic regulation of transcriptional states by chromatin and transcription factors. Nat Rev Genet 15, 69–81. 24342920 10.1038/nrg3623 PMC6322398

[tjp13073-bib-0132] Wang R , Asangani IA , Chakravarthi BV , Ateeq B , Lonigro RJ , Cao Q , Mani RS , Camacho DF , McGregor N , Schumann TE , Jing X , Menawat R , Tomlins SA , Zheng H , Otte AP , Mehra R , Siddiqui J , Dhanasekaran SM , Nyati MK , Pienta KJ , Palanisamy N , Kunju LP , Rubin MA , Chinnaiyan AM & Varambally S (2012). Role of transcriptional corepressor CtBP1 in prostate cancer progression. Neoplasia 14, 905–914. 23097625 10.1593/neo.121192 PMC3479836

[tjp13073-bib-0133] Wang S , Zeng H , Xie XJ , Tao YK , He X , Roman RJ , Aschner JL & Chen JX (2016). Loss of prolyl hydroxylase domain protein 2 in vascular endothelium increases pericyte coverage and promotes pulmonary arterial remodeling. Oncotarget 7, 58848–58861. 27613846 10.18632/oncotarget.11585 PMC5312280

[tjp13073-bib-0134] Ware KE , Hinz TK , Kleczko E , Singleton KR , Marek LA , Helfrich BA , Cummings CT , Graham DK , Astling D , Tan AC & Heasley LE (2013). A mechanism of resistance to gefitinib mediated by cellular reprogramming and the acquisition of an FGF2‐FGFR1 autocrine growth loop. Oncogenesis 2, e39. 23552882 10.1038/oncsis.2013.4 PMC3641357

[tjp13073-bib-0135] Wee S , Dhanak D , Li H , Armstrong SA , Copeland RA , Sims R , Baylin SB , Liu XS & Schweizer L (2014). Targeting epigenetic regulators for cancer therapy. Ann N Y Acad Sci 1309, 30–36. 24571255 10.1111/nyas.12356

[tjp13073-bib-0136] Welsh DJ , Peacock AJ , MacLean M & Harnett M (2001). Chronic hypoxia induces constitutive p38 mitogen‐activated protein kinase activity that correlates with enhanced cellular proliferation in fibroblasts from rat pulmonary but not systemic arteries. Am J Respir Crit Care Med 164, 282–289. 11463602 10.1164/ajrccm.164.2.2008054

[tjp13073-bib-0137] Wilkins MR (2018). Apoptosis signal‐regulating kinase 1 inhibition in pulmonary hypertension. Too much to ASK? Am J Respir Crit Care Med 197, 286–288. 28930481 10.1164/rccm.201709-1814ED

[tjp13073-bib-0138] Williams SM , Golden‐Mason L , Ferguson BS , Schuetze KB , Cavasin MA , Demos‐Davies K , Yeager ME , Stenmark KR & McKinsey TA (2014). Class I HDACs regulate angiotensin II‐dependent cardiac fibrosis via fibroblasts and circulating fibrocytes. J Mol Cell Cardiol 67, 112–125. 24374140 10.1016/j.yjmcc.2013.12.013 PMC4120952

[tjp13073-bib-0139] Wong N , Ojo D , Yan J & Tang D (2015). PKM2 contributes to cancer metabolism. Cancer Lett 356, 184–191. 24508027 10.1016/j.canlet.2014.01.031

[tjp13073-bib-0140] Wright JL , Zhou S , Preobrazhenska O , Marshall C , Sin DD , Laher I , Golbidi S & Churg AM (2011). Statin reverses smoke‐induced pulmonary hypertension and prevents emphysema but not airway remodeling. Am J Respir Crit Care Med 183, 50–58. 20709821 10.1164/rccm.201003-0399OC

[tjp13073-bib-0141] Young KC , Torres E , Hatzistergos KE , Hehre D , Suguihara C & Hare JM (2009). Inhibition of the SDF‐1/CXCR4 axis attenuates neonatal hypoxia‐induced pulmonary hypertension. Circ Res 104, 1293–1301. 19423843 10.1161/CIRCRESAHA.109.197533 PMC2757744

[tjp13073-bib-0142] Yu L & Hales CA (2011). Effect of chemokine receptor CXCR4 on hypoxia‐induced pulmonary hypertension and vascular remodeling in rats. Respir Res 12, 21. 21294880 10.1186/1465-9921-12-21 PMC3042398

[tjp13073-bib-0143] Yung LM , Nikolic I , Paskin‐Flerlage SD , Pearsall RS , Kumar R & Yu PB (2016). A selective transforming growth factor‐β ligand trap attenuates pulmonary hypertension. Am J Respir Crit Care Med 194, 1140–1151. 27115515 10.1164/rccm.201510-1955OC PMC5114445

[tjp13073-bib-0144] Zaret KS & Carroll JS (2011). Pioneer transcription factors: establishing competence for gene expression. Genes Dev 25, 2227–2241. 22056668 10.1101/gad.176826.111 PMC3219227

[tjp13073-bib-0145] Zhang H , Wang D , Li M , Plecita‐Hlavata L , D'Alessandro A , Tauber J , Riddle S , Kumar S , Flockton AR , McKeon BA , Frid MG , Reisz JA , Caruso P , El Kasmi KC , Jezek P , Morrell NW , Hu CJ & Stenmark KR (2017). The metabolic and proliferative state of vascular adventitial fibroblasts in pulmonary hypertension is regulated through a microRNA‐124/PTBP1 (polypyrimidine tract binding protein 1)/pyruvate kinase muscle axis. Circulation 136, 2468–2485. 28972001 10.1161/CIRCULATIONAHA.117.028069 PMC5973494

[tjp13073-bib-0146] Zhao L , Chen CN , Hajji N , Oliver E , Cotroneo E , Wharton J , Wang D , Li M , McKinsey TA , Stenmark KR & Wilkins MR (2012). Histone deacetylation inhibition in pulmonary hypertension: therapeutic potential of valproic acid and suberoylanilide hydroxamic acid. Circulation 126, 455–467. 22711276 10.1161/CIRCULATIONAHA.112.103176 PMC3799888

